# Review of Bone Modifying Agents in Metastatic Breast Cancer

**DOI:** 10.7759/cureus.13332

**Published:** 2021-02-13

**Authors:** Charumathi Raghu Subramanian, Swapna Talluri, Sanjana Mullangi, Manidhar R Lekkala, Bahar Moftakhar

**Affiliations:** 1 Department of Medicine, Washington Hospital Healthcare System, Fremont, USA; 2 Department of Medicine, Guthrie Robert Packer Hospital, Sayre, USA; 3 Department of Medicine, Hillcrest Medical Center, Tulsa, USA; 4 Department of Hematology/Oncology, James P Wilmot Cancer Institute, University of Rochester School of Medicine and Dentistry, Rochester, USA

**Keywords:** bone modifying agents, metastatic breast cancer

## Abstract

Bone is the most common site for distant metastases in breast cancer and can cause significant morbidity and mortality. Bone modifying agents (BMAs) that include bisphosphonates (BPAs) and denosumab help in decreasing and delaying skeletal-related events (SREs) associated with metastatic breast cancer. BPAs approved for use by the Food and Drug Administration (FDA) in bone metastases (BM) in the United States are pamidronate and zolendronic acid, while clodronate and ibandronate are licensed for use in other countries. Current American Society of Clinical Oncology (ASCO) guidelines recommend denosumab 120 mg subcutaneously every four weeks, or zolendronic acid 4 mg every four weeks or every 12 weeks, or intravenous pamidronate 90 mg every four weeks. Current guidelines do not recommend one BMA over another, however, zolendronic acid and denosumab were the most commonly used BMAs in population-based studies. Side effects of BMAs include acute phase reactions, hypocalcemia, nephrotoxicity, osteonecrosis of jaw, etc. While other side effects are common with both BPAs and denosumab, the latter has less nephrotoxic potential and is preferred for use in patients with renal failure. Current ASCO guidelines recommend continuing BMAs indefinitely, however, in clinical practice, this decision needs to be individualized, especially since there is no data on the impact of long-term use of BMAs. Further studies would need to be developed to develop an algorithm of SRE risk assessment and to determine which patients would benefit from BMAs.

## Introduction and background

Bone is the most common site for distant metastases in breast cancer [1] and can occur in up to 58% of patients with advanced disease [2]. Metastatic spread is the leading cause of breast cancer-related mortality. In this manuscript, we review the current data on the usage of bone modifying agents (BMAs) in metastatic breast cancer.

## Review

Mechanism of bone metastases (BM)

BM can be osteolytic, osteoblastic, or mixed, with the majority being osteolytic in nature [3]. Osteoclast activation is the main mechanism for osteolytic metastases associated with breast cancer. When osteoclasts are activated, multiple signaling cascades are turned on that destabilize the bone matrix, leading to accelerated bone loss and creating a favorable environment for tumor growth. BMAs, such as bisphosphonates (BPAs) and receptor activator of nuclear factor kappa-B ligand (RANKL) inhibitors (e.g., denosumab), modulate osteoclastic activity to suppress these effects [4].

BM in breast cancer

BM can cause significant morbidity and mortality in patients with breast cancer and, without BMAs, 50% of these patients can develop a skeletal-related event (SRE) [5]. SREs include hypercalcemia, spinal cord compression, pathological fracture, or the requirement of radiation or surgery for an impending fracture [5]. While women with bone-only metastatic breast cancer have a median survival of two-three years, this could be shortened to three months (hypercalcemia), four months (spinal cord compression), or 12 months (fracture), depending on the type of SRE [1,5].

Current BMAs in use for metastatic breast cancer are BPAs and denosumab. BPAs delay the onset of skeletal complications by inhibiting osteoclast-mediated bone resorption [6]. They bind to the bone mineral where they are absorbed by mature osteoclasts and subsequently induce osteoclast apoptosis [7]. Denosumab is a fully human monoclonal antibody that binds and inhibits the cytokine RANKL (receptor activator of NFκB ligand) which blocks osteoclast maturation and survival, thus reducing bone resorption [7].

BMAs used in metastatic breast cancer

The earliest studies of BPAs use for BM involved trials of clodronate [8] and pamidronate [9,10] in the early 1990s. Since then, several BPAs have been studied in various randomized controlled trials (RCTs). The BPAs pamidronate and zoledronic acid were approved for the treatment of BM by the Food and Drug Administration (FDA) in 1996 and 2002, respectively. Denosumab was approved by the FDA in 2010 [11], while clodronate and ibandronate have been licensed for use in other countries [12]. BPAs are administered either intravenously (zoledronic acid, pamidronate, ibandronate) or orally (clodronate or ibandronate). Denosumab is administered subcutaneously.

Current American Society of Clinical Oncology (ASCO) guidelines recommend BMAs for patients with breast cancer with BM who have evidence of bone destruction. The recommended agents for use are denosumab 120 mg subcutaneously every four weeks, intravenous pamidronate 90 mg over no less than 2 hours, or zoledronic acid 4 mg over no less than 15 minutes given every three to four weeks or every 12 weeks [13]. The recommendation for de-escalation of the frequency of zoledronic acid to every 12 weeks was added in 2017 after three RCTs (CALBG, ZOOM, and OPTIMIZE-2) and a meta-analysis by Ibrahim et al. showed no difference in outcomes between a shorter versus longer interval between infusions [14-17]. Various RCTs have demonstrated the efficacy of BMAs in metastatic breast cancer patients and in a recent large meta-analysis, BPAs showed a reduction in SREs by 16%-17% and decreased median time to SREs compared to placebo. Denosumab reduced SREs by 22% when compared to BPAs. None of the agents seemed to affect survival [18].

Comparison between different BMAs

Current guidelines do not recommend one BMA over another, however, in clinical practice, some agents are favored over others. Among BPAs, zoledronic acid is preferred compared to others due to efficacy data [19]. In head to head comparisons, zoledronic acid was found to be superior to ibandronate in preventing SREs in the ZICE trial [20]. In another RCT, zoledronic acid demonstrated higher efficacy in patients with breast carcinoma than pamidronate. In a multiple-event analysis, zoledronic acid significantly lowered the mean skeletal morbidity rate, increased the median time to first SRE, and reduced the risk of developing an SRE by 30% compared to pamidronate [21]. Another advantage of zoledronic acid over pamidronate is its shorter infusion time.

Among other bisphosphonates, ibandronate has also been widely studied. It is found to be more cost-effective compared to the more commonly used IV bisphosphonates, and this can be a consideration in select patients [22]. A comparison between oral and IV ibandronate showed equal efficacy [23]. Although oral treatment is more convenient, it should be used with caution given the potential for gastrointestinal (GI) side effects and lower efficacy in preventing SREs than the other IV agents [24].

When comparing BPAs to the RANKL inhibitor, denosumab, head to head trials were more favorable towards denosumab [25-27]. Stopeck et al. showed that denosumab reduced the risk of developing multiple SREs by 23% compared to zoledronic acid. Denosumab was also superior to zoledronic acid in reducing the mean skeletal morbidity rate (defined as the ratio of the number of SREs per patient divided by the patient’s time at risk) [26]. Denosumab had a more favorable safety profile with less incidence of renal toxicity and acute phase reactions. Convenient subcutaneous injection and no requirement for renal monitoring with denosumab are also cited as advantages [25,26]. Two different meta-analyses also demonstrated denosumab to be preferable over zoledronic acid and pamidronate [3,12].

In population-based studies, zoledronic acid and denosumab were the most commonly used BMAs [28]. Given the above data, some authors have questioned the guidelines not recommending one BMA over another, opining that denosumab seems to be superior to other BMAs in various aspects [29].

Side effects

Adverse events (AEs) with BMAs have been reported in trials and population studies (Table 1). These include GI-related events such as nausea and vomiting, as well as impaired renal function, acute phase reactions, electrolyte abnormalities, and osteonecrosis of jaw. Most AEs were low grade and in general, grade 3/4 AEs were rare [18].

**Table 1:** Side effects of bone modifying agents (BMAs) and monitoring parameters

Drug	Potential Side effects	Monitoring parameters	
		Prior to initiation of therapy	Prior to each dose
IV Zoledronic Acid	Hypocalcemia Influenza like reaction Musculoskeletal Pain Osteonecrosis of Jaw Atypical bone fractures Ocular Side effects	Dental Exam and appropriate preventative dentistry	Basic Metabolic Panel Calcium Phosphate Magnesium
IV Pamidronate	Electrolyte abnormalities Musculoskeletal Pain Atypical bone fractures Myelosuppression Osteonecrosis of Jaw	Dental Exam and appropriate preventative dentistry	Basic Metabolic Panel Calcium Phosphate Magnesium CBC
Ibandronate PO	GI side effects Hypocalcemia Influenza like reaction Musculoskeletal Pain Osteonecrosis of Jaw Atypical bone fractures Ocular Side effects	Dental Exam and appropriate preventative dentistry	Basic Metabolic Panel Calcium Phosphate Magnesium
Denosumab SQ	Dermatitis, rash, eczema Hypersensitivity Hypocalcemia Infections Musculoskeletal pain Atypical femur fractures Osteonecrosis of Jaw	Dental Exam and appropriate preventative dentistry	Basic Metabolic Panel Calcium Phosphate Magnesium Signs/Symptoms of hypersensitivity Signs/symptoms of dermatitis

Acute-phase reactions are defined as flu-like symptoms including pyrexia, chills, flushing, bone pain, and myalgias. These events generally occur within three days of treatment and subside rapidly thereafter [26,30]. Hypocalcemia is the most common electrolyte abnormality associated with BMA therapy [30]. This has been documented to be more frequent with denosumab than zoledronic acid in comparative clinical trials [25,26]. While most hypocalcemia events are asymptomatic and grade 2 or less, some could be severe, and even fatal cases have been reported [30,31]. Use of calcium and vitamin D supplements has been associated with a 40% lower risk of developing hypocalcemia with denosumab and it is recommended that patients receive daily supplementation. Serum calcium and vitamin D should be checked and be within normal limits prior to the initial dose and then checked prior to each dose thereafter [32,33]. However, if hypocalcemia develops, the BMA should be stopped and the patient should be treated accordingly [30,34].

Nephrotoxicity is another well-known AE of BPAs. Both zoledronic acid and pamidronate have been associated with renal failure. There have been more reports of renal failure with zoledronic acid than pamidronate [21,34]. Although acute renal failure may be reversible, some degree of irreversible impairment may persist leading to chronic renal failure. Treatment is generally not recommended if serum creatinine is > 3.0 mg/dl or creatinine clearance is < 30 ml/min [34]. Treatment should be withheld temporarily if serum creatinine increases greater than twice the patient’s baseline or greater than 0.5 mg/dl for patients with baseline ≤1.4 mg/dl and >1 mg/dl for others [35]. Zoledronic acid can be resumed when serum creatinine returns within 10% of baseline, however, should be permanently discontinued if no improvement is seen within four-eight weeks [35]. In contrast, denosumab was reported to have less renal toxicity in comparative trials [26,36]. Since denosumab elimination is not reliant on renal function, it presents a therapeutic option for patients with chronic renal failure [26]. Patients with chronic renal disease are at risk of severe hypocalcemia with denosumab compared to patients with normal renal function and this would need to be monitored closely [36].

Osteonecrosis of the jaw (ONJ) is another well recognized adverse event related to both bisphosphonates and denosumab [30]. A meta-analysis of several randomized trials did not show a significant difference in the incidence of ONJ between bisphosphonates and denosumab [37]. Poor oral hygiene, dentures, dentoalveolar surgery, dental and periodontal infections, systemic factors such as smoking, diabetes, renal insufficiency, total dose, and duration of treatment have been implicated as risk factors [30,38]. It is recommended that patients undergo a dental screening exam prior to the initiation of BMAs so that any required dental procedures can be carried out in advance [34]. While on BMAs patients should maintain good oral hygiene and have dental examinations at regular intervals. It is advised to avoid dental procedures while on BMAs, however, if it is deemed necessary, then it is prudent to stop BMAs prior to the procedure and resume only after appropriate wound healing [30,34].

Effect on quality of life (QoL)/pain management

Despite the benefit of reducing and delaying the onset of SREs, none of the BMAs have shown a survival benefit. In patients with metastatic and incurable disease, a relevant and important aim is improved QoL, a key component of which is pain control. Weinfurt et al. showed that women with metastatic breast cancer treated with BPAs had a gradual increase in physical, functional, and emotional well-being. Subjects also reported that a previous SRE was one of the reasons for the lack of improvement in QoL [39].

Porta-Sales et al. noted that BMAs while having only a weak analgesic effect, are actually beneficial in delaying the onset of bone pain [40]. Denosumab had a significant effect on pain, and patients treated with denosumab had more pain-free periods compared to patients treated with zoledronic acid [41]. Most patients (79%) had bone pain at the time of diagnosis of BM in a study by von Moos et al., and this was the main reason for initiating BMA [28]. Evidence suggests that early detection and treatment of BM before the onset of pain could benefit not only with pain relief but also decreased SREs [42]. BMAs’ analgesic effects however are modest and per ASCO guidelines, should not be used as the primary means of pain control.

When to start therapy/duration

The incidence of SREs is highest during the first year after the diagnosis of metastatic breast cancer. A Danish-based population study estimated that the cumulative incidence of SREs in patients with metastatic breast cancer was 38.5% at one year and 51.7% at five years [43]. Appropriately, in recent population-based studies, most patients with BMs were treated with BMAs early in their treatment course [28].

Current ASCO guidelines recommend continuing treatment with BMAs indefinitely [44] unless toxicity occurs or patient preference changes. If a breakthrough SRE occurs while on treatment with a BMA, it is generally advised to continue BMAs as they can lengthen the time to subsequent SREs [21,45]. There are no current guidelines for changing from one BMA to another. The patient could either continue with the same BMA or consider a switch. Denosumab could be a reasonable alternative if the patient was on an IV bisphosphonate earlier since it has been proven to be successful in patients previously treated with IV BPAs [25].

In clinical practice, the decision to treat indefinitely would need to be individualized. Several studies of real-world data show that patients experienced fewer SREs than patients enrolled on RCTs. This is thought to be due to a large proportion of patients who have both visceral and BM, and unlike in the bone-only disease population, as seen in these studies, the former have shorter survival time and thus less opportunity for SREs or fractures in their lifespan [5,46]. Gainford et al. also observed that patients in clinical practice do not undergo serial imaging if they are asymptomatic in contrast to patients in RCTs who undergo X-rays at regular intervals [5]. This was also elucidated in other population studies where around 30% of patients developed clinically significant SREs, while in RCTs, these numbers were in the 40s and 50s (treatment and placebo arms respectively) [1,47]. Further studies will be necessary to develop an algorithm of SRE risk assessment and to determine which patients would benefit from BMAs.

Age >60 years, previous SRE, a predominance of osteolytic lesions, and brief pain inventory score > 3 units are some of the parameters identified in previous studies that have demonstrated an increased risk of SRE [48]. Machine learning models to predict SREs are also being studied, and if developed, could be used in metastatic breast cancer cases [49].

Future studies/trials

The ongoing trials, REDUSE and REaCT-BTA, are studying different dosing intervals of denosumab (four weeks vs twelve weeks) and results are currently being awaited [13]. There are no RCTs to determine the duration of treatment for BMAs. There is a need for these RCTs as well as a need for more data on the impact of long-term use of BMAs.

## Conclusions

In conclusion, BMAs play an important role in the treatment of breast cancer patients with BM. Based on RCT results and real-world data, zoledronic acid and denosumab are favored compared to other BMAs. While denosumab has shown slight superiority in RCTs in reducing the rate of SREs and delaying the onset of SREs, zoledronic is also safe, efficacious, and importantly, more cost-effective than the former. Denosumab is preferred in patients with chronic renal failure or those who are also receiving nephrotoxic chemotherapeutic agents. As described above, more research is needed to identify patients who would benefit from BMAs, to determine the optimal duration of treatment, and to elucidate long-term effects.
